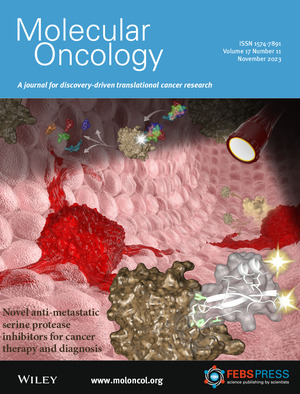# Issue Information

**DOI:** 10.1002/1878-0261.13241

**Published:** 2023-11-01

## Abstract

The cover schematic depicts how serine peptidase based agents target tumors and inhibit metastatic spread. Protease mediated targeting of mesotrypsin and KLK6 via human amyloid β‐protein precursor Kunitz protease inhibitor domain (APPI) could potentially be used for cancer therapy and imaging. Read the full article by Amiram Sananes *et al*. in pp. 2337–2355.

Illustration credit: Dr. Itay Cohen.